# Evaluation of the Effects of Mulberry Leaf Extracts *Morus alba* L. on Cardiovascular, Renal, and Platelet Function in Experimental Arterial Hypertension

**DOI:** 10.3390/nu17010049

**Published:** 2024-12-27

**Authors:** Masoud Akbari Aghdam, Ana Pagán, Joaquín García-Estañ, Noemí M. Atucha

**Affiliations:** Departamento Fisiología, Facultad Medicina, Instituto Murciano de Investigación Biosanitaria, Universidad de Murcia, 30120 Murcia, Spainanapagan@um.es (A.P.); ntma@um.es (N.M.A.)

**Keywords:** arterial hypertension, nitric oxide, *Morus alba* extract (MAE), platelet aggregation, blood pressure

## Abstract

Introduction: Numerous epidemiological studies have demonstrated that consuming foods rich in polyphenols and flavonoids can have beneficial effects on various diseases, including arterial hypertension (HTN). Recent research from our laboratory has shown that certain flavonoids exhibit antihypertensive properties in several animal models of HTN. Our objective was to evaluate the effect of *Morus alba* L. (white mulberry) extracts in an experimental HTN model characterized by nitric oxide (NO) deficiency. Methods: Male Sprague-Dawley rats were divided into four groups: a control group, hypertensive rats treated with an NO synthesis inhibitor (L-NAME) in drinking water for six weeks, L-NAME rats treated with *Morus alba* L. extract, and L-NAME rats treated simultaneously with captopril. After six weeks of treatment, we measured blood pressure, endothelial vascular function in the aorta, and platelet aggregation function. Results: *Morus alba* L. extract partially prevented the development of arterial hypertension due to NO deficiency, although it did not completely normalize blood pressure as captopril did. The extract reduced the excessive vasoconstrictor response to phenylephrine in aortic rings and improved vasodilation in response to acetylcholine, with both effects dependent on increased NO production. *Morus alba* L. extract also reduced the increased platelet aggregation in response to ADP and collagen in hypertensive animals, although it did not fully normalize this function. Conclusions: *Morus alba* L. extract demonstrates antihypertensive effects, improves vascular reactivity, and reduces platelet aggregation in a model of arterial hypertension. These effects are primarily related to an increase in nitric oxide activity.

## 1. Introduction

Arterial hypertension is a major global public health challenge and a leading cause of cardiovascular and renal diseases [1,2]. The primary approach to treating hypertension involves lifestyle modifications, including dietary changes, increased physical activity, and weight reduction [3]. A reduction in blood pressure by 5 mm Hg can significantly decrease the risk of stroke by 34% and ischemic heart disease by 21%, while also reducing cardiovascular disease mortality [4].

In recent years, there has been a growing focus on identifying bioactive components in foods and diets for the treatment and prevention of hypertension [5]. *Morus alba* L., commonly known as white mulberry, is cultivated in eastern countries, with its leaves traditionally used for silkworm cultivation. Various parts of the mulberry plant, from root bark to leaves, have been extensively studied for their health benefits, including antioxidant, hypolipidemic, antihyperglycemic, antiatherogenic, antiviral, antimicrobial, and neuroprotective effects [6,7,8,9,10,11,12]. Animal studies have shown that ingestion of mulberry leaves can normalize abnormally elevated blood pressure [11,13]. Long-term treatment with mulberry leaves has been found to significantly restore impaired blood vessel reactivity, including decreased dilation and increased constriction, to normal levels [9]. These effects may be attributed to the inhibition of the angiotensin-converting enzyme, resulting in reduced angiotensin II levels. Another potential mechanism for the antihypertensive action could be the presence of γ-aminobutyric acid (GABA) in mulberry leaves [11].

Previous studies from our laboratory, focusing on two animal models of arterial hypertension, have linked the antihypertensive effects of several flavonoid-rich extracts to an increase in nitric oxide levels [14,15]. Building on this research, the present study aims to analyze the cardiovascular, vascular, platelet, and renal effects of an ethanolic extract of *M. alba* leaves in an experimental model of arterial hypertension induced by nitric oxide deficiency. This model is particularly suitable for testing substances that may increase NO production and reduce oxidative stress, as these are two key mechanisms underlying L-NAME-induced hypertension. By inhibiting nitric oxide synthase, L-NAME treatment leads to decreased NO bioavailability and increased reactive oxygen species (ROS) production, resulting in endothelial dysfunction and vasoconstriction. Furthermore, this model allows for the direct assessment of a compound’s ability to counteract the specific pathophysiological changes associated with NO deficiency, including increased NADPH oxidase activity and reduced antioxidant capacity. Thus, the L-NAME hypertension model provides an ideal platform to evaluate the potential of *M. alba* extract (MAE) in restoring NO-dependent vascular function and mitigating oxidative-stress-induced cardiovascular alterations.

## 2. Materials and Methods

### 2.1. Morus alba L. Extracts

Three different types of *M. alba* (white mulberry) leaf extracts were prepared for this study. Two were ethanolic extracts, using 50% and 70% ethanol as extracting agents, while the third was an aqueous extract using ultrapure water. The *M. alba* variety was selected from the IMIDA Germplasm Bank (BAGERIM) based on its demonstrated antioxidant effects in previous studies with *Caenorhabditis elegans* and its anti-inflammatory effects in obese mice [16]. Mulberry leaves were collected from the selected variety from the IMIDA germplasm collection, washed, carefully dried, and then lyophilized until use. The extraction process started from the freeze-dried mulberry leaves, and the extracts were prepared by incubating them while shaking for 15 min at RT and alternating with 15 min sonication, with each type of extracting agent as appropriate and always protected from light. The samples were then centrifuged at 8000× *g* and the supernatant was filtered, followed by concentration and removal of ethanol using a rotary evaporator at 40 °C. As the last step of the extraction, all of the extracts prepared were subjected to freeze-drying and stored at −20 °C until use. Preliminary experiments showed that all of these extracts exhibited no toxicity in L929 cell cultures and demonstrated both anti-inflammatory capacity and good antioxidant activity. The alcoholic extract made with 70% ethanol was chosen as the extract to be used in the animal studies due to its studied biocompatibility and low toxicity in addition to the potential of this solvent to extract more active compounds from leaf samples.

### 2.2. Animals

The experiments were conducted using male Sprague-Dawley rats, bred and raised in the animal facilities of the Universidad de Murcia. The rats were housed in a temperature-controlled environment with a 12:12 h light–dark cycle at the Animal Care Facility of the University of Murcia (REGA ES300305440012). All animal care and experimental procedures adhered to the guidelines established by the European Union for the protection of animals used in scientific research (86/609/EEC). The study protocol was reviewed and approved by the Animal Care and Use Committee of the University of Murcia (C1310050303), ensuring compliance with ethical standards for animal experimentation.

### 2.3. Experimental Groups

**Figure 1:**
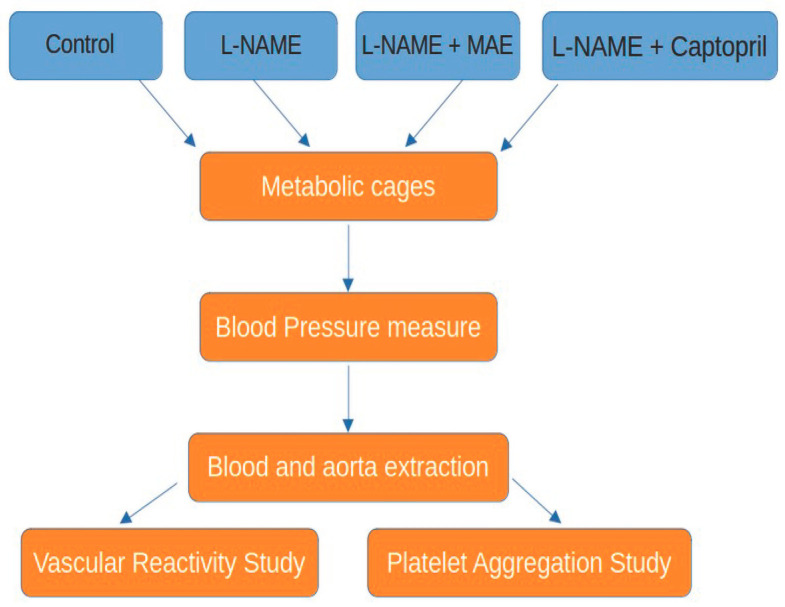
Summary of the experimental design.

Rats (8-week-old) were randomized into four groups: 1. control (n = 8), rats without any treatment; 2. L-NAME (n = 8), rats receiving chronic L-NAME for 6 weeks (N-w-nitro-l-arginine methyl ester, 10 mg/kg/day); 3. *M. alba* extract (MAE, n = 8), rats simultaneously treated with L-NAME plus the MAE extract (100 mg/kg/day given as gavage); and 4. captopril (n = 8), rats simultaneously treated with L-NAME plus captopril (an inhibitor of angiotensin-converting enzyme) at a dose of 100 mg/kg/day added to the drinking water. Figure 1 summarizes the experimental design.

### 2.4. Experimental Procedures

Rats were initially kept in their regular cages for 4–5 weeks. During this period, they were gradually acclimated to individual metabolic cages (Tecniplast, Radnor, PA, USA) for two days each week. In the sixth week, after a two-day adaptation period, 24 h measurements of food and water intake, as well as urinary volume, were conducted. Urine samples were collected, centrifuged (1000× *g*, 10 min) to remove solid matter, and stored at −80 °C for subsequent analysis.

#### 2.4.1. Measurement of Blood Pressure and Blood Extraction

After completing the metabolic study, the animals were anesthetized with sodium pentobarbital (5 mg/Kg, i.p.) and placed on a heated table to maintain a body temperature of 37 °C. Blood pressure was measured with an indwelling catheter into the right femoral artery, as previously described [14,15]. After that, blood was collected for platelet aggregation studies, as previously published [17]. Then, the animal was euthanized by opening the thorax, and the descending thoracic aorta was extracted and placed in a Petri dish containing oxygenated and pre-warmed Krebs solution for vascular reactivity studies.

#### 2.4.2. Vascular Reactivity Study

The method used to analyze vascular reactivity has been previously published in detail [14,15]. Briefly, the aorta was cleaned, cut into four rings, and mounted in organ baths (organ bath system LE 01004, Panlab, Barcelona, Spain) containing physiological Krebs solution maintained at 37 °C and continuously bubbled with 95% O_2_ and 5% CO_2_. The aortic tension was measured with isometric force transducers (TRI202P, Panlab) and acquired using an AD Instrument system (Oxford, UK). The rings were equilibrated for at least 45 min at a resting tension of 2 g before experiments began. The experimental protocol was as follows:

- A cumulative dose–response curve to phenylephrine (Phe, 10^−9^–10^−4^ mol/L) was performed.
- After washing and re-stabilization, the rings were pre-contracted with a submaximal dose of Phe (10^−6^ mol/L).
- A cumulative dose–response curve to acetylcholine (Ach, 10^−9^–10^−4^ mol/L) was performed to assess endothelium-dependent vasodilatation.
- After washing and re-stabilization, the rings were incubated with the NOS-inhibitor L-NAME (10^−4^ M) for 30 min.
- Another cumulative concentration–response curve to Ach was performed to evaluate the role of NO in endothelium-dependent vasodilatation.
- Finally, a single dose of sodium nitroprusside (SNP, 10^−4^ M) was added to test endothelium-independent vasodilator responses.

Responses to Phe are expressed in grams, while relaxation to Ach and SNP is expressed as a percentage of the maximal Phe effect. All reagents and vasoactive compounds were purchased from Sigma-Aldrich (St. Louis, MO, USA) and Panreac (Barcelona, Spain).

#### 2.4.3. Platelet Aggregation Study

The method used to analyze vascular reactivity has been previously published in detail [17]. In brief, after centrifuging blood to obtain platelet-rich plasma, platelets were resuspended in Hepes buffer without Ca^2+^, and the platelet concentration adjusted to 2 × 10^8^ platelets/mL using a hematology analyzer. Platelets were kept at 19 °C in a thermostated bath, and Ca^2+^ was added immediately before the start of the experiments. Platelet aggregation responses were analyzed using a 2-channel optical aggregometer (Chronolog, model 700, Chronolog Corporation, Havertown, PA 19083, USA). Two platelet agonists were used to assess the aggregation response of the prepared platelet samples:

- Adenosine diphosphate (ADP) at concentrations of 0.75, 1.25, 2.5, 5, 10, and 20 µM.
- Collagen at concentrations of 0.75, 1.25, 2.5, and 5 µM.

### 2.5. Reagents

The compounds, their sources, and the purity of the reagents are as follows:

- ADP, Sigma-Aldrich; ACS grade, HPLC > 95%.
- Collagen, Hart, >95% type-I fibrils from equine tendon.
- L-NAME, Thermo scientific (Waltham, MA, USA), HPLC > 97.5%.
- Sodium nitroprusside, Sigma-Aldrich; ACS grade, >99%.
- Reagents used for Krebs solution or in ring experiments or in platelets: Sigma-Aldrich; Bioperformance-certified (BioUltra or BioXtra grades).
- Ethanol absolute; vWR; grade TechniSolv, >99.5%.

### 2.6. Statistical Analysis

Results are presented as arithmetic means with standard deviations. Statistical analyses were conducted as follows: for vascular reactivity studies, within-group differences were assessed using analysis of variance (ANOVA) for multiple comparisons, with Duncan’s test applied to identify differences between pairs when significant results were obtained. Contractile responses to phenylephrine are expressed in grams, while relaxation responses to acetylcholine are presented as percentages of phenylephrine-induced contraction. The 50% effective dose (ED50) values were calculated through regression analysis for each ring individually, and between-group differences in ED50 values were assessed using Student’s *t*-test. For platelet aggregation studies, the area under the curve (AUC) and the slope of responses were calculated individually, with between-group differences analyzed using two-way ANOVA for multiple comparisons and post hoc Duncan tests performed when necessary. For other between-group comparisons, one-way analysis of variance was employed. A *p*-value < 0.05 was considered statistically significant for all analyses.

## 3. Results

**Figure 2:**
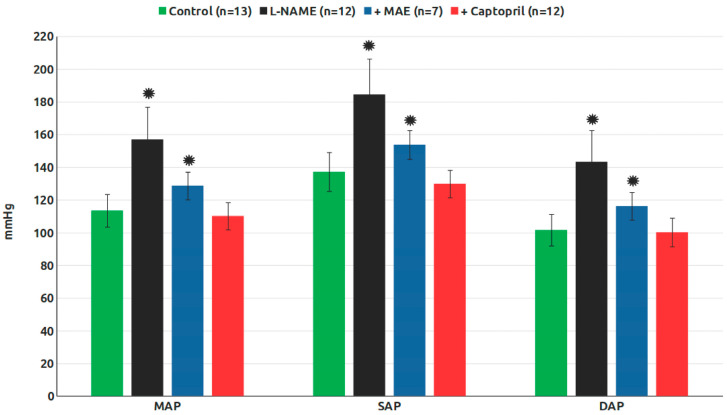
Blood pressure in the experimental groups (MAP, mean arterial pressure; SAP, systolic arterial pressure; DAP, diastolic arterial pressure). *, *p* < 0.05 vs. control group.

Figure 2 illustrates the mean arterial pressure (MAP), the systolic blood pressure (SBP), and the diastolic blood pressure (DBP) across the experimental groups. Chronic L-NAME treatment induced a significant increase in all three parameters. Concurrent administration of MAE or captopril significantly reduced these elevated pressures, although the MAE-treated group did not fully return to control values.

**Figure 3:**
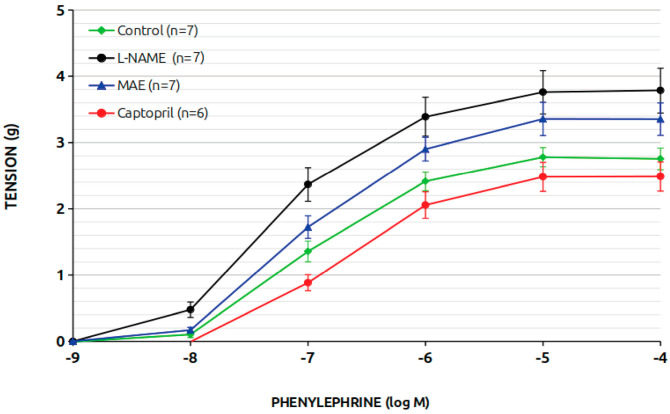
Reactivity to phenylephrine in aortic rings.

Figure 3 depicts the vasoconstrictor response of aortic rings to phenylephrine. The L-NAME-treated group exhibited an enhanced response compared to the control group. While simultaneous MAE treatment reduced this response, it remained higher than in the control animals. Captopril treatment effectively diminished the vasoconstrictor response.

Table 1 presents the effective dose 50 (ED50 in mM), pD2 (-M), and maximum response (g) data. These values were elevated in hypertensive animals, while the *M. alba* extract treated group showed lower but still significantly higher values compared to the control and captopril groups.

**Figure 4:**
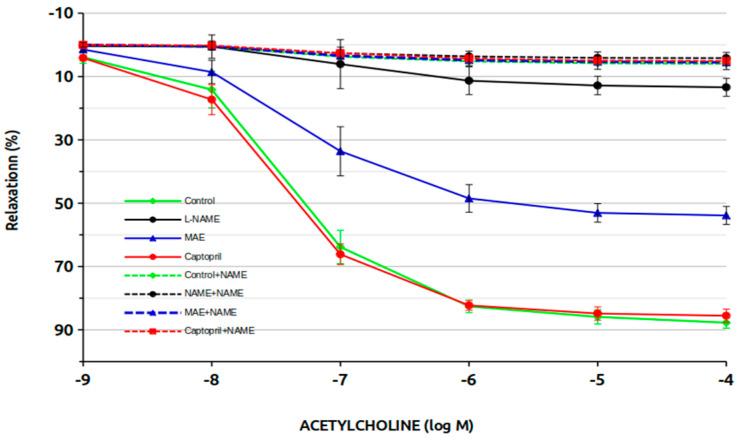
Vasodilator response to acetylcholine in aortic rings.

Figure 4 and Table 2 illustrate the vasodilatory response to acetylcholine. Control and captopril groups demonstrated the highest vasodilatory response (approximately 90%), while the L-NAME group showed only a 10% response. MAE treatment in L-NAME-treated animals markedly improved the vasodilatory response to nearly 60%. In chronically L-NAME-treated groups, the vasodilatory response was almost completely abolished. The beneficial effect of MAE was attributed to nitric oxide (NO) involvement, as it was eliminated when L-NAME was acutely added to the rings. This response is more evident when examining the ED50 or pD2 data, with statistically significant differences observed between the L-NAME group treated with MAE and the untreated L-NAME group. The vasodilatory response to sodium nitroprusside at the experiment’s conclusion was preserved at nearly 100% with no significant differences between groups.

**Figure 5:**
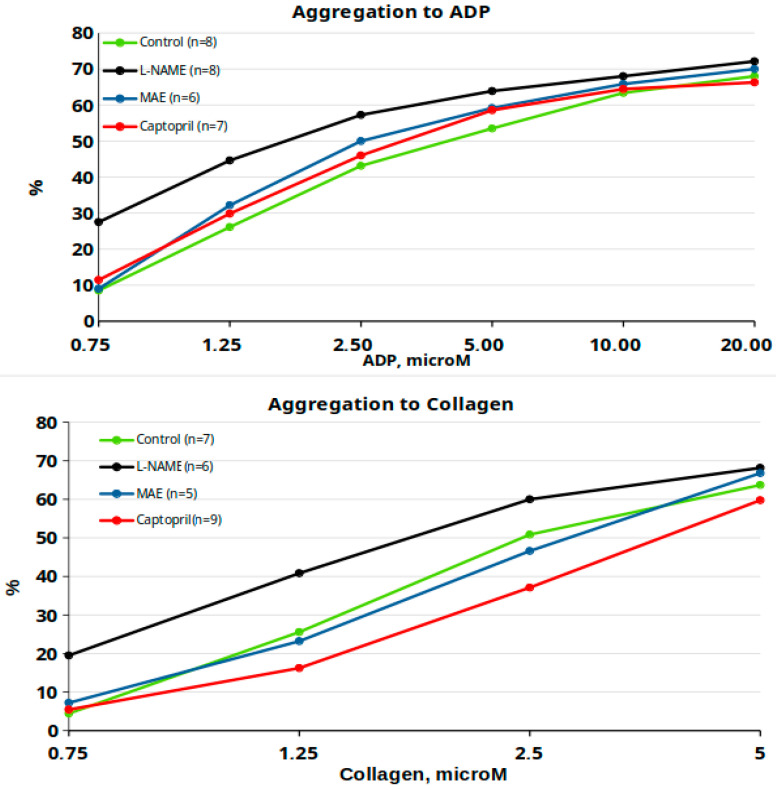
Platelet aggregation to ADP and collagen.

Figure 5 illustrates the platelet aggregation percentages in response to ADP and collagen across different animal groups. While the maximum aggregation values are similar among groups, significant differences in overall responses are evident, as confirmed by ANOVA results. Similar patterns were observed in the area under the curve and slope analyses. Rather than conducting point by point statistical comparisons, which might yield numerous results of limited clinical relevance, this study focused on calculating ED50 values for each group to assess overall response changes. This approach provides a more clinically meaningful interpretation of the data. The key findings from the ED50 analysis show that the group treated chronically with MAE exhibited a significantly lower ED50 compared to the control group (*p* < 0.05), while no other significant differences were observed in ED50 values among the other groups.

## 4. Discussion

In recent years, our laboratory has focused on studying the role of flavonoids in an experimental model of arterial hypertension induced by nitric oxide (NO) deficiency through the administration of L-NAME, an inhibitor of NO synthesis. Previous studies have shown that lemon extract, grapefruit extract, cocoa extract, and apigenin exhibit moderate antihypertensive effects in animals with L-NAME-induced hypertension [14,15]. While the causes of spontaneous hypertension are multifactorial [17,18]—encompassing genetics, the sympathetic nervous system, and the renin–angiotensin system—the primary cause of L-NAME-induced hypertension is a reduction in NO production, which indirectly leads to overactivation of vasoconstrictor mechanisms, such as the renin–angiotensin system and increased oxidative stress [19]. These mechanisms are often cited as targets for the beneficial effects of flavonoids. In our previous work, we observed significant effects of flavonoids on blood pressure as well as renal and vascular function. With this background, we decided to evaluate the effects of another flavonoid-rich compound, *Morus alba* L. extract (MAE), using the well-established L-NAME hypertension model. The aim of this study was to assess the vascular and renal effects of MAE in this experimental model of arterial hypertension caused by NO synthesis inhibition.

The treatment with MAE was well-tolerated by the animals, similarly to other treatments we have tested, with no adverse effects observed. Regarding blood pressure—one of the primary outcomes analyzed—chronic treatment with L-NAME significantly increased the mean arterial pressure (MAP), systolic blood pressure (SBP), and diastolic blood pressure (DBP), consistent with previous findings from our group. Notably, treatment with MAE significantly reduced MAP, indicating that MAE largely prevented the hypertensive effects induced by L-NAME, although blood pressure levels remained slightly higher than those in the control group. This suggests that MAE can partially mitigate arterial hypertension caused by NO deficiency. In comparison, chronic treatment with captopril almost completely prevented hypertension, consistent with earlier studies from our laboratory. The antihypertensive effect of captopril is primarily attributed to its inhibition of angiotensin II formation, a key mechanism driving hypertension and its associated complications.

Another objective of this study was to evaluate vascular reactivity in aortic rings. As expected, the vasoconstrictor response in L-NAME-treated animals was significantly higher than in control animals, consistent with both our previous data and findings from the literature [14,15]. This heightened response is due to reduced NO production and the consequent loss of vasodilatory protection against increased vascular tone. Interestingly, simultaneous treatment with MAE reduced this heightened vasoconstrictor response, although it remained higher than that observed in control animals. This suggests that MAE may increase NO levels in aortic rings. Treatment with captopril effectively normalized the vasoconstrictor response, as observed in previous studies by our group. The effects of MAE were further supported by ED50 and maximum response data, which showed reductions compared to the L-NAME group but did not reach normal control values. Additional evidence for MAE’s potential role in enhancing NO production was observed during vasodilation experiments with acetylcholine. In L-NAME-treated animals, acetylcholine-induced vasodilation was nearly abolished (13% of control levels), whereas animals treated with MAE achieved a maximum vasodilation response of 54%. Importantly, these differences were eliminated when L-NAME was acutely added to aortic rings during experiments, confirming that MAE improves vascular NO production. As expected, sodium-nitroprusside-induced vasodilation remained intact across all groups, indicating no direct effect on smooth muscle function and ensuring experimental reliability.

Regarding platelet aggregation, chronic L-NAME treatment increased platelet aggregating responses. Treatment with MAE significantly improved these responses but did not fully normalize them compared to controls. Captopril treatment normalized platelet aggregation responses, particularly at lower agonist doses. Similar trends were observed when analyzing the area under the curve and slope data for aggregation responses. Interestingly, only the group treated chronically with MAE exhibited a lower ED50 compared to controls; however, these values still did not reach those observed in control animals. These findings suggest that MAE reduces excessive platelet aggregation induced by chronic L-NAME treatment, a beneficial effect that could improve platelet function and vascular responses under conditions of heightened aggregation. However, it remains unclear whether this effect is directly related to increased NO production or another mechanism.

To our knowledge, there are no other studies using the *M. alba* extract in the model of arterial hypertension through nitric oxide deficit. However, several studies have demonstrated the potential of MAEs in treating hypertension through mechanisms that could be relevant to L-NAME hypertension [20,21]. For instance, MAE has been shown to evoke endothelial vasorelaxation through a nitric-oxide-dependent pathway and to increase endothelial nitric oxide synthase (eNOS) phosphorylation [22]. Moreover, in vivo administration of MAE reduced blood pressure levels in wild-type mice but not in eNOS-deficient mice [23]. This suggests that the antihypertensive effect of MAE is mediated through eNOS, which could be relevant in L-NAME hypertension.

## 5. Conclusions

In conclusion, MAE exhibits antihypertensive effects and improves vascular reactivity and platelet aggregation in an experimental model of arterial hypertension caused by nitric oxide deficiency. These beneficial effects appear to be closely related to enhanced nitric oxide activity [14,15].

## Figures and Tables

**Table 1:** Aortic reactivity to phenylephrine.

Group	ED50	pD2	Max
Control	0.499 ± 0.002	6.302 ± 0.002	2.791 ± 0.145
L-NAME	0.517 ± 0.006 *	6.288 ± 0.005 *	3.793 ± 0.55 *
MAE	0.508 ± 0.003 *	6.294 ± 0.003 *	3.372 ± 0.316 *
Captopril	0.496 ± 0.003	6.304 ± 0.003	2.495 ± 0.176

Data are mean ± standard deviation. *, *p* < 0.05 vs. control.

**Table 2:** Aortic relaxation to acetylcholine.

Group	ED50	pD2	Max
Control	8.379 ± 0.518	5.079 ± 0.026	87.723 ± 1.767
L-NAME	0.743 ± 0.067 *	6.143 ± 0.042 *	13.786 ± 4.771 *
MAE	2.794 ± 0.224 *	5.562 ± 0.032 *	54.154 ± 3.623 *
Captopril	7.834 ± 0.416	5.110 ± 0.024	85.539 ± 1.642

Data are mean ± standard deviation. *, *p* < 0.05 vs. control.

## Data Availability

The data presented in this study are available on request from the corresponding author.
